# A Highly Efficient *In Vitro* Regeneration System for Pearl Millet (*Pennisetum glaucum*) Variety “Shandaweel-1” Using Immature Inflorescences

**DOI:** 10.3390/plants15142126

**Published:** 2026-07-09

**Authors:** Amira K. Mohamed, Ashraf H. Fahmy, Walid M. Fouad

**Affiliations:** 1 Graduate Program of Biotechnology, School of Science and Engineering, The American University in Cairo, New Cairo 11835, Egypt; amirakhaled@aucegypt.edu; 2 The Agriculture Genetic Engineering Research Institute, Giza 12619, Egypt; ashrafbiotechnology@gmail.com; 3 Department of Biological, Chemical & Global Health Sciences, School of Science and Engineering, The American University in Cairo, New Cairo 11835, Egypt

**Keywords:** pearl millet, somatic embryogenesis, *in vitro* regeneration, tissue culture, immature inflorescences

## Abstract

Pearl millet (*Pennisetum glaucum*) is a widely cultivated C4 cereal crop in tropical and subtropical regions, serving as a food and feed source in developing countries across Africa and Asia. Despite its agronomic importance, research efforts aimed at developing improved millet varieties under the current climatic changes remain limited, particularly in Africa, including Egypt. This study aimed to establish an efficient regeneration system for the “Shandaweel-1” Egyptian pearl millet variety using immature inflorescences as explants. Six different callus induction media (CIM) treatments were evaluated for their effects on somatic embryogenesis, callus type, vitrification rate, and regeneration efficiency. Among the tested treatments, Murashige and Skoog (MS) media additionally supplemented with 1.0 mg L^−1^ of l-proline, 1.0 mg L^−1^ of l-asparagine, 10 mg L^−1^ of silver nitrate (AgNO_3_), 0.32 mg L^−1^ of copper sulfate (CuSO_4_), 1.0 mg L^−1^ of 2,4-dichlorophenoxyacetic acid (2,4-D), and 0.5 mg L^−1^ of 6-benzylaminopurine (BAP) produced a high callogenesis rate (93%) and the highest regeneration efficiency (47.6%). The combination of l-proline and l-asparagine enhanced the callus quality and regeneration potential more effectively than casein hydrolysate alone, whereas AgNO_3_ addition did not have any significant impact on the vitrification or callogenesis rates. This study represents the first successful establishment of an *in vitro* regeneration system for an Egyptian pearl millet variety, providing a valuable platform for future genetic modification aimed at enhancing stress resilience and crop productivity.

## 1. Introduction

Pearl millet (*Pennisetum glaucum*) is one of the major millets belonging to the cereal family *Poaceae*. It is an annual C4 monocot crop widely cultivated in arid and semi-arid regions [1]. Pearl millet is the sixth most cultivated cereal crop within the *Poaceae* family following maize, wheat, rice, barley, and sorghum [2]. The largest producer of pearl millet is India, accounting for about 40% of its global production, followed by Niger and China [3]. It is also the fourth most cultivated crop in India following rice, wheat, and maize [4,5]. Pearl millet has recently gained increasing global attention, initiated by India’s designation of 2018 as the national year of millet [6] followed by the Food and Agriculture Organization (FAO)’s recognition of 2023 as the international year of millets. These initiatives emphasized the nutritional, environmental, and socioeconomic importance of millets and their contribution in achieving the Sustainable Development Goals (SDGs) [7]. Although it is known as an ancient crop, pearl millet is also considered a future crop due to its climate resilience properties [8,9,10,11]. Pearl millet displays remarkable tolerance to harsh environmental conditions including drought, heat, and poor soil fertility [12]. Pearl millet is a valuable dietary option as it is rich in proteins, essential minerals, dietary nutrients, and antioxidants, while also being gluten-free. Despite its beneficial nutritional profile and climate resilience, it is an underutilized resource compared to other cereals [13,14,15]. In Egypt, research on pearl millet has primarily focused on field assessments and yield evaluation under various environmental stresses to identify superior feed varieties [16]. While genetic improvement has been successfully implemented in cereal crops such as maize, wheat, and rice [17,18,19], the establishment of *in vitro* regeneration and genetic transformation systems has not yet been reported for Egyptian local pearl millet varieties [20]. Therefore, this study focuses on establishing an efficient *in vitro* regeneration system for the Egyptian local variety “Shandaweel-1” as an essential step for future genetic transformation and molecular crop improvement. This study investigates the potential of immature inflorescences as an explant source as well as the effect of different plant growth regulators (PGRs) and other media components on callus induction, callus quality, and subsequent shoot induction frequency of the produced callus.

## 2. Results

### 2.1. Overview of the In Vitro Regeneration System of Shandaweel-1 from Immature Inflorescences

**Figure 1:**
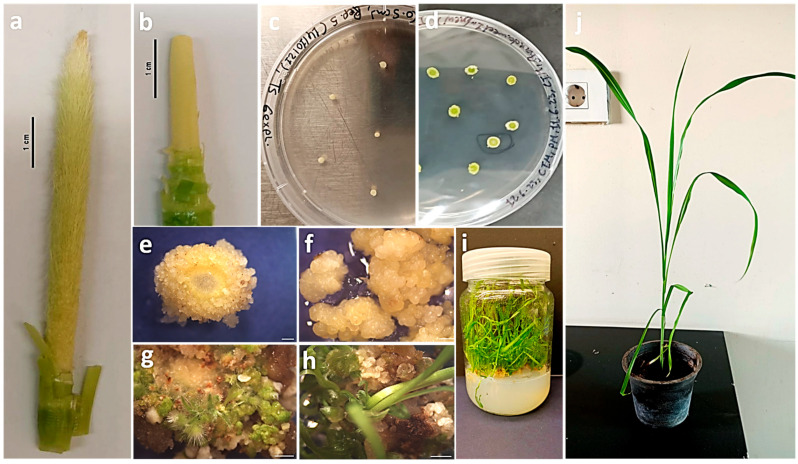
Overview of the *in vitro* regeneration system for pearl millet variety Shandaweel-1. Sterilized immature inflorescences before cutting with different sizes: (**a**) 7 cm, (**b**) 2 cm. Cultured immature inflorescences disks on callus induction media 5 (CIM5, as described in Section 4), driven from different lengths: (**c**) 0.5 cm, (**d**) 7.5 cm. (**e**,**f**) Yellow embryogenic callus generated on callus induction media 4 (CIM4, as described in Section 4). Calli during different regeneration stages: (**g**) two weeks and (**h**) eight weeks. (**i**) Elongated *in vitro* plants with extensive root formation in a glass culture jar. (**j**) Successfully acclimatized *in vitro* pearl millet plants after one month. Scale bar: 1 cm (**a**,**b**) and scale bar: 0.1 cm (**e**–**h**).

Preliminary experiments with immature inflorescences ranging in sizes from 0.2 cm to 4.5 cm and 5 cm to 10 cm did not show significant differences in callus induction frequencies. Therefore, immature inflorescences ranging from 0.2 to 10 cm in length were utilized as explants for callus initiation (Figure 1a,b). Surface sterilization using 80% commercial bleach and removal of the surrounding leaf whorls resulted in minimal contamination rates during culture establishment. Explant disks cultured on callus induction media (CIM; Figure 1c,d) generated yellow embryogenic compact (Figure 1e) and friable calli (Figure 1f). After 10 days on shoot induction media (SIM), embryogenic calli developed green sectors accompanied by shoot primordia formation, giving rise to small shoots (Figure 1g). Through the late phases of regeneration, shoots reached suitable lengths (≥3 cm) for subsequent stages (Figure 1h). During shoot elongation and rooting phases, regenerated plantlets demonstrated vigorous shoot extension and root formation (Figure 1i), reflecting the high regenerative capacity of immature inflorescences and their suitability for successful acclimatization (Figure 1j).

### 2.2. Effect of Culture Media on Callus Quality

**Figure 2:**
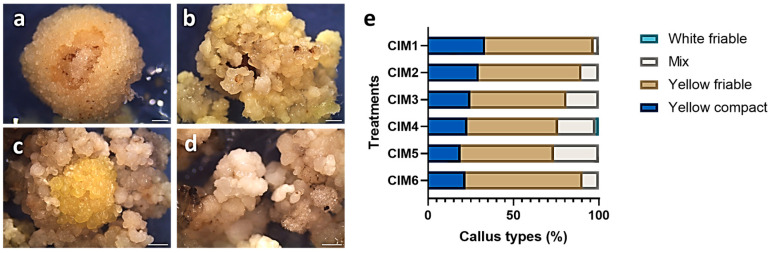
Assessment of diverse callus types across callus induction media treatments. (**a**) Yellow compact callus, (**b**) yellow friable callus, (**c**) mixed types of callus, (**d**) white friable callus, (**e**) percentage of each type across the six callus induction media. Scale bar: 0.1 cm.

Following six weeks of callus culture, all media treatments induced embryogenic callus from immature inflorescences. The resulting calli exhibited variations in color and texture, including yellow compact/friable, white friable, and mixed phenotypes (Figure 2). Compact calli were characterized by densely aggregated embryonic structures tightly associated with the rachis (Figure 2a). In contrast, friable calli consisted of loosely organized embryonic structures that readily separated from one another and from the rachis (Figure 2b). In comparison, mixed calli displayed combinations of both colors and textures (Figure 2c). White friable calli were less frequent and mainly appeared within mixed populations (Figure 2d). Alternatively, during subcultures, watery and browning calli were considered non-embryogenic due to progressive necrosis. The yellow friable callus was the predominant response across all treatments (Table 1). The highest proportion of yellow friable callus was recorded on callus induction media 6 (68.54%), whereas callus induction media 3 (CIM3) exhibited the lowest percentage (53%). Yellow compact callus represented the second most observed type, with frequencies ranging from 19.15% on CIM5 and 33.5% on CIM1, with an average of 25.3% across treatments. White friable callus was observed only on CIM4, where it accounted for 2% of the callus population. In contrast, mixed calli varied across treatments, with the highest frequency recorded on CIM5 (26.4%), followed by CIM4 (22%), while CIM1 demonstrated the lowest proportion (3%) (Figure 2e).

### 2.3. Effect of Culture Media on Vitrification Rates

**Figure 3:**
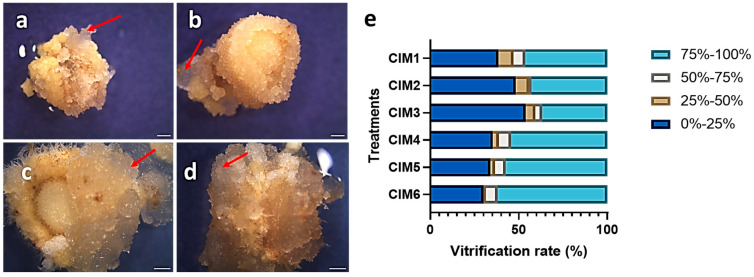
Assessment of vitrification levels during the callus induction phase. Visual scoring of the vitrification rates, indicated by the red arrows. (**a**) Vitrified callus within the lowest vitrification category (0–25%), (**b**) vitrified callus within the vitrification category 25–50%, (**c**) vitrified callus within the vitrification category 50–75%, (**d**) vitrified callus within the highest vitrification category (75–100%), (**e**) percentage of vitrification rates across the six callus induction media. Scale bar: 0.1 cm.

Vitrification was observed during callus induction as translucent watery callus, distinguished from the embryogenic calli, as shown in Figure 3. Based on the extent of the affected calli, vitrification severity was categorized into four ranges 0–25%, 25–50%, 50–75%, and 75–100%. Calli within the lowest vitrification category retained better structural integrity and clearer characteristics (Figure 3a); on the other hand, increasing vitrification progressively masked embryonic callus quality (Figure 3b–d). Across all six CIM treatments, the most abundant vitrification categories were 0–25% and 75–100% (Table 1). CIM3 exhibited the lowest vitrification severity (0–25%) with a frequency of 54%. On the contrary, CIM6 showed the highest vitrification severity (75–100%) with a frequency of 61.84% (Figure 3e). These findings indicated different vitrification responses among the tested treatments, suggesting the apparent impact of the explants and media treatments on callus quality.

### 2.4. Effect of Culture Media on Callogenesis and Regeneration Efficiency

**Figure 4:**
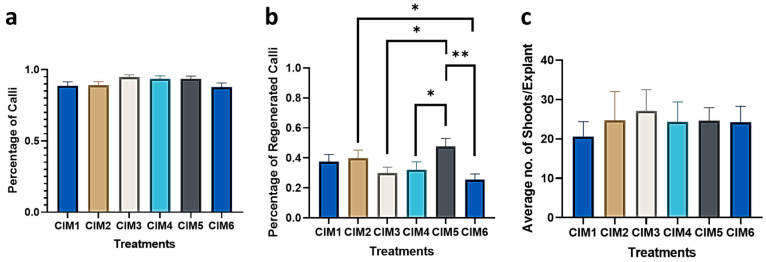
Impact of callus induction media treatments on Shandaweel-1 immature inflorescences’ callogenesis and regeneration efficiency. (**a**) Percentage of callogenesis rate per treatment with no statistical significance shown between the tested treatments. (**b**) Percentage of regeneration efficiency per treatment showing statistical significance between the tested treatments, using one-way Analysis of Variance (ANOVA) and compared by Fisher’s Least Significant Difference (LSD). (**c**) The average number of shoots per explant across treatments showing no statistical differences. * *p*-value < 0.05, ** *p*-value < 0.01.

Comparable callus induction frequencies were obtained from the six callus induction media treatments (CIM1–CIM6), ranging from 87% to 95% (Table 1). The highest callus induction percentage was recorded in CIM3 (95%), followed by CIM4 and CIM5 (93%), whereas CIM1, CIM2, and CIM6 showed induction frequencies of 88%, 89%, and 87%, respectively. No significant difference was detected among the tested treatments, indicating no notable effect of the explant length or media additives on callus formation (Figure 4a). However, significant differences were observed in the regeneration efficiency of the calli driven from the different treatments. CIM5 has shown the highest regeneration percentage of 47.6%, followed by CIM2 (39.8%), CIM1 (37.5%), and CIM4 (32%) (Table 1). Despite their high callogenesis rate, CIM3 and CIM6 have shown the lowest regeneration efficiencies at 29.8% and 25%, respectively. Significant differences were observed between CIM2/CIM5 and CIM6 (*p*-value < 0.05), highlighting the negative impact of increasing the 2,4-dichlorophenoxyacetic acid (2,4-D) concentration from 1 mg L^−1^ to 3 mg L^−1^ on the regeneration capacity. Moreover, the significant difference between CIM4 and CIM5 suggested that 6-benzylaminopurine (BAP) in combination with a lower 2,4-D concentration further enhanced the regeneration efficiency of calli (Figure 4b). Similarly, significant differences were detected between CIM5 and CIM3, indicating the positive effect of l-proline and l-asparagine in comparison to casein hydrolysate on regeneration. The average number of shoots regenerated per explant, presented in Table 1, did not differ significantly among treatments. CIM2, CIM4, CIM5, and CIM6 produced comparable average number of shoots (~24) per explant. CIM1 produced the lowest average number of shoots (~20) per explant, whereas CIM3 recorded the highest average number of shoots (~27) per explant (Figure 4c). Regenerated plants of “Shandaweel-1” with healthy shoot and root systems were successfully acclimatized under greenhouse conditions, reaching a survival rate of 93.3% after one month of the transfer.

## 3. Discussion

Suitable explant selection is a critical determinant for the success of any *in vitro* culture system. Immature inflorescences are more feasible and relatively stable compared to immature embryos in cereal tissue culture [21]. In the present study, immature inflorescences of the “Shandaweel-1” variety showed a high response rate in callus production and demonstrated efficient regeneration potential. Previous studies have similarly demonstrated the suitability of immature inflorescences as explant sources for pearl millet tissue culture [22,23,24]. The emergence of flag leaves after about 50 days of planting served as a reliable indicator for harvesting explants at the appropriate developmental stage, which is consistent with earlier reports describing the enclosure of immature inflorescences within the emerged flag leaves [25,26]. Considerable variations in immature inflorescence length were observed among the harvested explant material, likely reflecting genotype-dependent response to the environmental conditions. Shorter immature inflorescences were observed to produce better callus quality and less vitrification compared to longer explants. This is aligned with earlier findings in pearl millet tissue culture, where lengths ranging from 0.5 to 5 cm exhibited better response in tissue culture and were mostly used in immature inflorescence cultures [23,26,27,28,29]. Additionally, similar observations were reported with tissue culture of other cereals like sorghum [30], rice [31], maize [32], and wheat [33].

Somatic embryos were observed to arise from the axis and meristematic primordia of the florets and spikelet, and formed distinct embryogenic callus morphologies, including yellow and white compact and friable calli during callus culture establishment. These features resembled previously reported regenerable callus types, where embryogenic callus was described as a yellow, nodular, and highly proliferative mass of cells [22,28,29]. Vitrified calli were frequently observed during callus induction, particularly from relatively larger explants. This aligns with other reports where these tissues were observed around the callus mass [28,29]. Unlike non-embryogenic calli, these vitrified calli could be easily removed during subculture, without severely affecting the embryogenic callus growth. According to the literature, silver nitrate (AgNO_3_) decreases vitrification caused by ethylene accumulation in culture vessels, especially during the regeneration phase [34,35]. Additionally, it was reported to enhance embryogenesis and organogenesis in many cereals like wheat [36,37], barley [38], and sorghum [39]. In this study, 10 mg L^−1^ AgNO_3_ was added to test its effect on reducing hyperhydricity. This contrasted our findings where AgNO_3_ did not significantly affect callus production and vitrification or directly influence the regeneration rates. This comes in alignment with one report on pearl millet where AgNO_3_ did not significantly enhance regeneration and negatively impacted the rooting phase in certain genotypes [40].

Tissue culture media is a vital success factor of plant regeneration systems. It contains amino acids, macronutrients, and micronutrients required for plant development. Furthermore, it can be exogenously supplemented with plant growth regulators (PGRs) and other additives to improve the plant’s growth [41,42,43]. Callus induction media composition markedly influenced the regeneration performance of pearl millet explants. The addition of l-proline and l-asparagine amino acids was found to boost the regenerability of the explants despite not having an impact on callus formation. l-proline is an essential amino acid that acts as an osmoprotectant to the cells and is known for its positive impact on the *in vitro* regeneration of plants [44,45]. While l-asparagine is an important biostimulant [46], l-proline and L-asparagine likely enhance somatic embryogenesis through multiple overlapping mechanisms rather than acting solely as osmolytes or nitrogen sources. l-proline contributes to stress tolerance, redox homeostasis, and reactive oxygen species (ROS) regulation, thereby supporting the acquisition and maintenance of embryogenic competence [47,48]. It has also been associated with cell differentiation and development, and embryo formation [49], while l-asparagine primarily serves as a readily assimilable source of nitrogen that supports the high metabolic demands of rapidly dividing embryogenic cells, while also potentially influencing nitrogen signaling pathways and cellular reprogramming [46,50]. Previous studies reported the positive effect of l-proline, alone or in combination with other amino acid sources, on millet callus regeneration [40,51,52]. Additionally, it was found to enhance explant regeneration of other cereals including sorghum [39], maize [32], and wheat [53]. Another important additive that was included in all treatments is copper sulfate (CuSO_4_). Copper is a fundamental microelement that improves callus growth as well as shoot and root formation. Its addition was reported to improve the *in vitro* response of many cereal explants, including millets [54], wheat [55], rice [56], barley [57], and sorghum [58].

Plant growth hormones are the main key players for somatic embryogenesis and organogenesis, where the balance between auxin and cytokinin signaling becomes crucial for coordinating the transition from callus proliferation to embryo maturation and shoot regeneration [43,59]. The tested concentrations of the synthetic auxin 2,4-D (1, 2, 3 mg L^−1^) produced similar callogenesis frequencies, all exceeding 85%, which is higher than callogenesis rates of other studies on pearl millet, where only up to 50% callogenesis was obtained [23,25]. Yet, a carryover effect was noticed affecting regeneration efficiencies. The significantly lower regeneration efficiency observed in CIM6 in comparison to CIM5 may be attributed to the elevated concentration of 2,4-D (3 mg L^−1^). While 2,4-D is essential for callus induction and the acquisition of embryogenic competence, excessive concentrations can maintain cells in a highly proliferative and undifferentiated state, thereby delaying their progression toward embryo maturation and shoot formation and increasing the abnormalities in developing somatic embryos [60]. This was in alignment with reports where callus production and regeneration were significantly hindered by higher 2,4-D concentrations, while lower auxin concentrations were favored for pearl millet [29,61,62,63] and other cereal tissue culture [64,65]. This was in contrast with other studies where higher 2,4-D concentrations did not affect callus culture of certain varieties of pearl millet [66,67,68]. A combination of lower 2,4-D with the cytokinin BAP resulted in improved regeneration rates, in line with other pearl millet studies [35,62]. Meanwhile, omitting BAP in CIM4 reduced the regeneration potential of embryogenic explants, implying the importance of cytokinin incorporation in supporting callus regeneration. A higher concentration of BAP (1.5 mg L^−1^) was used in combination with 0.2 mg L^−1^ 1-naphthaleneacetic acid (NAA) in the regeneration media treatment, and it was shown to support shoot proliferation of the generated embryogenic callus. Supplementation of BAP in regeneration media was frequently used in pearl millet tissue culture research [23,28,69,70], while other reports have shown that incorporating cytokinin alone with no auxins during shoot induction was also efficient [29]. Although no significant differences in shoot production were observed among the tested treatments, the high regeneration capacity achieved in this study (≥20 shoots per explant), which exceeded previous reports of approximately six shoots per explant [28], is likely due to the genotype. Similar to other reports [22,70], promoting shoot and root elongation on ½ Murashige and Skoog (MS) hormone-free media treatment or with very low auxin concentrations was found to be effective in shoot elongation and root formation while encouraging gradual removal of residual exogenous hormones.

Given pearl millet’s high nutritional value and resilience to harsh environmental conditions, pearl millet represents a promising target for genetic improvement, particularly in developing countries like Egypt. Despite its considerable potential, pearl millet remains an underutilized crop receiving comparatively limited research attention. Therefore, greater emphasis should be placed on advancing research and promoting its agricultural importance globally and locally, particularly in the context of climate resilience, sustainable agriculture, and food security. The regeneration platform established in this study provides a foundation for genetic transformation and genome-editing approaches aimed at enhancing key agronomic traits, including drought tolerance, heat stress resilience, and improved nutrient-use efficiency. Future research should also investigate the molecular mechanisms underlying embryogenic competence and regeneration potential, particularly through the characterization of key somatic embryogenesis-related regulators such as *BABY BOOM* (*BBM*), *WUSCHEL* (*WUS*), and *LEAFY COTELYDON* (*LEC1/2*) [71]. Furthermore, the application of emerging cellular-resolution techniques, including single-cell transcriptomics, could enable identification of cell type-specific regulatory networks governing somatic embryogenesis and regeneration [72]. Such future studies would enhance our understanding of the regulatory pathways underlying regeneration and accelerate the development of more efficient tissue culture and transformation systems of pearl millet.

## 4. Materials and Methods

Establishment of Donor Plant Material: Seeds of landrace variety “Shandaweel-1” were obtained from the Agriculture Research Center (ARC), Giza, Egypt. Three plantation cycles were initiated in the open field at the Agriculture Genetic Engineering Research Institute (AGERI) located in the ARC, during the spring and summer seasons (April, May, June). Plant thinning was done at a young stage of growth, and plants were irrigated weekly. The emergence of flag leaves after 40 to 60 days was an indication of an appropriate phase for harvesting immature inflorescences.

Establishment of Aseptic Cultures: Leaf whorls containing immature inflorescences were thoroughly washed under tap water to remove any surface debris. Under aseptic conditions, leaf whorls containing immature inflorescences were sterilized using 70% ethanol for two minutes, followed by 80% commercial bleach and two drops of Tween-20 for 30 min. The leaf whorls were subsequently rinsed and washed six to eight times with sterilized distilled water for five minutes per wash.

*In vitro* Media Preparation: All culture media components, including MS basal salts, sucrose, amino acids, and PGRs, were purchased from Phytotechnology Laboratories (Overland Park, KS, USA) except for the casein hydrolysate, l-asparagine, and copper sulfate (CuSO_4_), which were purchased from Sigma-Aldrich (Saint Louis, MO, USA). MS basal salts, sucrose, and amino acids were dissolved in one liter of distilled water, according to the formulations shown in Table 2, followed by pH adjustment to 5.8 using potassium hydroxide prior to the addition of agar. The media treatments were sterilized by autoclaving for 20 min at 121 °C. After cooling down to 65 °C, filter-sterilized PGRs, CuSO_4_, and Silver Nitrate (AgNO_3_) were supplemented to the MS media (Table 2). The AgNO_3_ was prepared in its active form of silver thiosulfate (STS) [Ag(S_2_O_3_)_2_]^3−^. Media were poured into sterile Petri dishes (94 × 16 mm) purchased from Greiner Bio-One (Greiner Bio-One, Kremsmünster, Austria) for callus induction and regeneration phases, while autoclaved glass jars were used for shoot elongation and rooting media.

*In Vitro* Callus Culture Establishment and Regeneration of Immature Inflorescences: Sterilized immature inflorescences ranging between 0.2 and 10 cm were aseptically removed from the surrounding leaf sheath and sectioned into 1–2 mm disks prior to culturing on the six CIM treatments shown Table 2. A minimum of 50 explant disks were initiated per treatment. The first two treatments (CIM1 and CIM2) were aimed at testing the impact of AgNO_3_ on callogenesis and vitrification rates. CIM3 was meant to assess the impact of casein hydrolysate on callus induction. While CIM4 to CIM6 tested the impact of PGRs, where CIM4 had cytokinin omitted, CIM5 and CIM6 included different concentrations of the synthetic auxin 2,4-D (1 and 3 mg L^−1^). The cultured disks were incubated in a dark growth chamber adjusted to 25 ± 2 °C. Callus formation was initiated for three weeks before subculturing on the same treatments. The explants were maintained on CIM for a total of six weeks before moving them to the shoot induction media (SIM), containing 10 mg L^−1^ AgNO_3_, 1.5 mg L^−1^ BAP, and 0.2 mg L^−1^ NAA (Table 2). The regeneration phase was carried out in a growth chamber with a 16/8 h photoperiod, 2800 lux intensity, and adjusted to 27 ± 1 °C. The explants spent a total of six to eight weeks in the regeneration phase with a subsequent subculture every two weeks on fresh media. Regenerated plantlets were moved to ½ MS hormone-free shoot elongation media (SEM) (Table 2) for shoot elongation and root formation and were subcultured every two weeks for two months prior to acclimatization. Candidate plantlets with healthy and sturdy shoot and root systems were picked for acclimatization in the greenhouse. After cleaning the roots of the clones from media particles, the roots were dipped in fungicide and planted into small-sized pots filled with a 2:1 mixture of sand and peat moss and were covered with transparent polyethylene plastic bags. After 10 days, openings were introduced on the plastic bags, followed by another week before removing the plastic bags. When plants reached an appropriate length, they were transferred to larger-sized pots to provide better space for elongation and root extension. Survival rates of the acclimatized plants were recorded after one month of acclimatization.

Data collection and Statistical Analysis: The above experiments were repeated three times. For each replicate, data items, such as the number of explants generating callus, distribution of callus types, vitrification rate (assessed as the vitrified portion per each callus clump and scored based on severity), the number of callus-generating shoots (regenerated callus), and the number of shoots per explant, were collected at the end of each phase. Data analysis was performed on the clean compiled data of the repeated experiments after outlier removal. Calli and regenerated plantlets were imaged using Leica dissecting microscope (Wetzlar, Germany) equipped with LAS EZ software, version 3.4.0 [73]. The number of acclimatized plants was recorded and reported as a percentage after one month of acclimatization. Data analysis was performed using GraphPad Prism, version 8.4.3,(Boston, MA, USA), by one-way Analysis of Variance (ANOVA), compared by Fisher’s least significant difference (LSD), to assess statisitical significance among treatments, shown by the *p*-value [74].

## 5. Conclusions

The first efficient *in vitro* regeneration system was successfully developed for the Egyptian variety “Shandaweel-1”. Immature inflorescences as an explant source displayed high embryogenic competence and regeneration potential. Although the tested callus induction treatments did not significantly influence the callogenesis rate, a carryover effect was observed during the regeneration phase. Among the tested treatments, the medium containing a low concentration of 2,4-D (1.0 mg L^−1^) in combination with BAP (0.5 mg L^−1^), as well as l-proline, l-asparagine, and AgNO_3_ (10 mg L^−1^), produced high-quality callus, with a callus induction rate of 93% and the highest regeneration frequency of 47.6% with an average of 24 shoots per explant. *In vitro* pearl millet plants were successfully acclimated under greenhouse conditions with a success rate of 93.3%. The establishment of a reliable regeneration system for an Egyptian pearl millet variety represents an important step towards the development of genetic transformation systems for local accessions. This tissue culture system provides a foundation for genetic improvement and introduction of agronomically valuable traits. Such advancements are relevant for arid and semi-arid regions including Egypt, where climate change, water scarcity, and food insecurity increasingly challenge agricultural sustainability. Given pearl millet’s high nutritional value and climate resilience, it holds considerable potential for supporting sustainable agriculture and food security in Egypt.

## Figures and Tables

**Table 1:** Relationship between callus quality characteristics and regeneration responses of immature inflorescences of pearl millet variety “Shandaweel-1” initiated on the six callus induction media (CIM) treatments.

Treatment	Predominant Callus Type	Predominant Vitrification Range	Callus Induction (%)	Regeneration Frequency (%)	Mean Number of Shoots per Regenerated Explant
CIM1	Yellow friable (63.50%)	75–100% vitrification (46.25%)	88.0	37.50 abc	20
CIM2	Yellow friable (60.02%)	0–25% vitrification (48.36%)	89.0	39.80 ab	24
CIM3	Yellow friable (53.00%)	0–25% vitrification (54.00%)	95.0	29.80 bc	27
CIM4	Yellow friable (53.00%)	75–100% vitrification (54.32%)	93.0	32.00 bc	24
CIM5	Yellow friable (54.45%)	75–100% vitrification (57.29%)	93.0	47.60 a	24
CIM6	Yellow friable (68.54%)	75–100% vitrification (61.81%)	87.0	25.00 c	24

Values represent treatment means. No significant differences were observed among tested treatments for callus induction frequency or mean shoot number per explant. Within the regeneration frequency column, means followed by different letters are significantly different according to one-way ANOVA (*p* ≤ 0.05) compared by LSD.

**Table 2:** Composition of callus induction (CIM), shoot induction (SIM), and shoot elongation (SEM) media per liter for *in vitro* regeneration of “Shandaweel-1” immature inflorescences.

	CIM1	CIM2	CIM3	CIM4	CIM5	CIM6	SIM	SEM
MS Basal Salts	4.33 g	4.33 g	4.33 g	4.33 g	4.33 g	4.33 g	4.33 g	2.165 g
Sucrose	30.0 g	30.0 g	30.0 g	30.0 g	30.0 g	30.0 g	30.0 g	30.0 g
l-Proline	1.0 g	1.0 g	-	1.0 g	1.0 g	1.0 g	1.0 g	-
l-Asparagine	1.0 g	1.0 g	-	1.0 g	1.0 g	1.0 g	1.0 g	-
Casein Hydrolysate	-	-	1.0 g	-	-	-	0.5 g	-
Agar	8.0 g	8.0 g	8.0 g	8.0 g	8.0 g	8.0 g	8.0 g	8.0 g
MS Vitamins	100.0 mg	100.0 mg	100.0 mg	100.0 mg	100.0 mg	100.0 mg	100.0 mg	100.0 mg
* CuSO_4_	0.32 mg	0.32 mg	0.32 mg	0.32 mg	0.32 mg	0.32 mg	0.32 mg	0.32 mg
AgNO_3_	-	10.0 mg	10.0 mg	10.0 mg	10.0 mg	10.0 mg	10.0 mg	-
2,4-D	2.0 mg	2.0 mg	2.0 mg	2.0 mg	1.0 mg	3.0 mg	-	-
BAP	0.5 mg	0.5 mg	0.5 mg	-	0.5 mg	0.5 mg	1.5 mg	-
NAA							0.2 mg	-

* CuSO_4_ was supplemented in addition to the Cupric Sulfate•5H_2_O already present in the MS basal salts.

## Data Availability

The original contributions presented in this study are included in the article. Further inquiries can be directed to the corresponding author.

## Abbreviations

The following abbreviations are used in this manuscript:

FAO	Food and Agriculture Organization
SDGs	Sustainable Development Goals
CIM	Callus Induction Media
SIM	Shoot Induction Media
SEM	Shoot Elongation Media
MS	Murashige and Skoog
2,4-D	2,4-dichlorophenoxyacetic acid
BAP	6-benzylaminopurine
NAA	1-Naphthaleneacetic acid
CuSO_4_	Copper Sulfate
AgNO_3_	Silver Nitrate
STS	Silver Thio Sulfate
ANOVA	Analysis of Variance
LSD	Fisher’s Least Significant Difference
ROS	Reactive Oxygen Species
*BBM*	*BABY BOOM*
*WUS*	*WUSCHEL*
*LEC1/2*	*LEAFY COTELYDON 1/2*
